# Transgenic Expression of miR-133a in the Diabetic Akita Heart Prevents Cardiac Remodeling and Cardiomyopathy

**DOI:** 10.3389/fcvm.2019.00045

**Published:** 2019-04-24

**Authors:** Tyler N. Kambis, Hamid R. Shahshahan, Sumit Kar, Santosh K. Yadav, Paras K. Mishra

**Affiliations:** ^1^Department of Cellular and Integrative Physiology, University of Nebraska Medical Center, Omaha, NE, United States; ^2^Department of Anesthesiology, University of Nebraska Medical Center, Omaha, NE, United States

**Keywords:** fibrosis, P-V loop, collagenase, SERCA-2a, metabolic remodeling, hypertrophy, Oil-O Red, zymography

## Abstract

Advanced diabetes mellitus (DM) may have both insulin resistance and deficiency (double DM) that accelerates diabetic cardiomyopathy (DMCM), a cardiac muscle disorder. Reduced cardiac miR-133a, a cardioprotective miRNA, is associated with DMCM. However, it is unclear whether increasing miR-133a levels in the double DM heart could prevent DMCM. We hypothesized that increasing cardiac levels of miR-133a could prevent DMCM in Akita, a mouse model of double DM. To test the hypothesis, we created Akita/miR-133aTg mice, a new strain of Akita where miR-133a is overexpressed in the heart, by crossbreeding male Akita with female cardiac-specific miR-133a transgenic mice. We validated Akita/miR-133aTg mice by genotyping and phenotyping (miR-133a levels in the heart). To determine whether miR-133a overexpression could prevent cardiac remodeling and cardiomyopathy, we evaluated cardiac fibrosis, hypertrophy, and dysfunction (P-V loop) in 13–15 week male WT, Akita, Akita/miR-133aTg, and miR-133aTg mice. Our results revealed that miR-133a overexpression in the Akita heart prevents DM-induced cardiac fibrosis (reduced collagen deposition), hypertrophy (decreased beta-myosin heavy chain), and impaired contractility (downregulated calcium handling protein sarco-endoplasmic reticulum-ATPase-2a). These results demonstrate that increased levels of miR-133a in the DM heart could prevent cardiac remodeling. Our P-V loop analysis showed a trend of decreased cardiac output, stroke volume, and ± dp/dt in Akita, which were blunted in Akita/miR-133aTg heart. These findings suggest that 13–15 week Akita heart undergoes adverse remodeling toward cardiomyopathy, which is prevented by miR-133a overexpression. In addition, increased cardiac miR-133a in the Akita heart did not change blood glucose levels but decreased lipid accumulation in the heart, suggesting inhibition of metabolic remodeling in the heart. Thus, miR-133a could be a promising therapeutic candidate to prevent DMCM.

## Introduction

Diabetic cardiomyopathy (DMCM) is a diabetes mellitus (DM)-induced cardiac muscle disorder independent of hypertension, coronary artery disease, or valvular disease that leads to heart failure (1, 2). DM increases the risk of heart failure (3–5), which is not decreased by the intensive glycemic control in DM patients (6–8). Thus, a novel therapeutic approach is needed to decrease the risk of DM-induced heart failure. With increasing awareness toward DM, more people are diagnosed with early-stage DM. The risk of developing cardiovascular diseases in these patients are comparatively higher than non-diabetic patients (9). Presently, the major strategy to treat these patients are controlling the blood glucose levels. However, this strategy may not revert/cure adverse cardiac remodeling or DMCM. DMCM is a multifactorial disease thus targeting a specific gene/protein may not ameliorate DMCM. Regulating several genes in a biological network to restore cardiac homeostasis could be a therapeutic strategy to prevent adverse cardiac remodeling and DMCM.

MicroRNAs (miRNAs) are a novel class of conserved, endogenous, regulatory RNAs that micromanage several genes in a biological network by translational repression or mRNA degradation (10, 11). The majority of biological processes in the body are regulated by miRNAs, which have emerged as a potential therapeutic target for cardiovascular diseases (12). There are more than 2,000 miRNAs present in humans (13), and nearly 800 miRNAs present in the human heart (14). Only a dozen miRNAs are differentially expressed in the failing heart. miR-133a is one of the downregulated miRNAs in the failing heart (15). It is the most abundant miRNA in the human heart (14), which protects the heart against adverse remodeling (16, 17). It is reduced in the DM hearts of humans (18) and rodents (19, 20). Although miR-133a mimic treatment mitigates DM-induced cardiac dysfunction in DM rats and mice (19, 21, 22), it is unclear whether increased miR-133a levels in the DM heart could prevent DMCM.

DMCM is a complex disease (23). Progression of DMCM depends on insulin deficiency (T1DM), insulin resistance (T2DM), and a combination of both insulin resistance and deficiency (double DM) (24). Different animal models are used to study DMCM; however, none of them perfectly mimic human DMCM. Akita mice are a spontaneous genetic model of T1DM, where insulin production is reduced due to mutation of insulin 2 (orthologous to human Insulin) gene (25). A mutation of the Insulin gene causes T1DM in humans (26). Thus, insulin 2 mutant (Ins2^+/−^) Akita is a genetic mouse model of T1DM relevant to humans. Notably, Akita mice also show insulin resistance, a feature of T2DM (27). Thus, Akita mice is a model of double DM, a condition often present in patients with advanced-stage DM (28). In the present study, we sought to investigate if miR-133a overexpression in the Akita heart could prevent DM-induced cardiac remodeling and DMCM.

## Results

### Generation of a New Strain of Akita Mice With Transgenic miR-133a Expression in the Heart

**Figure 1:**
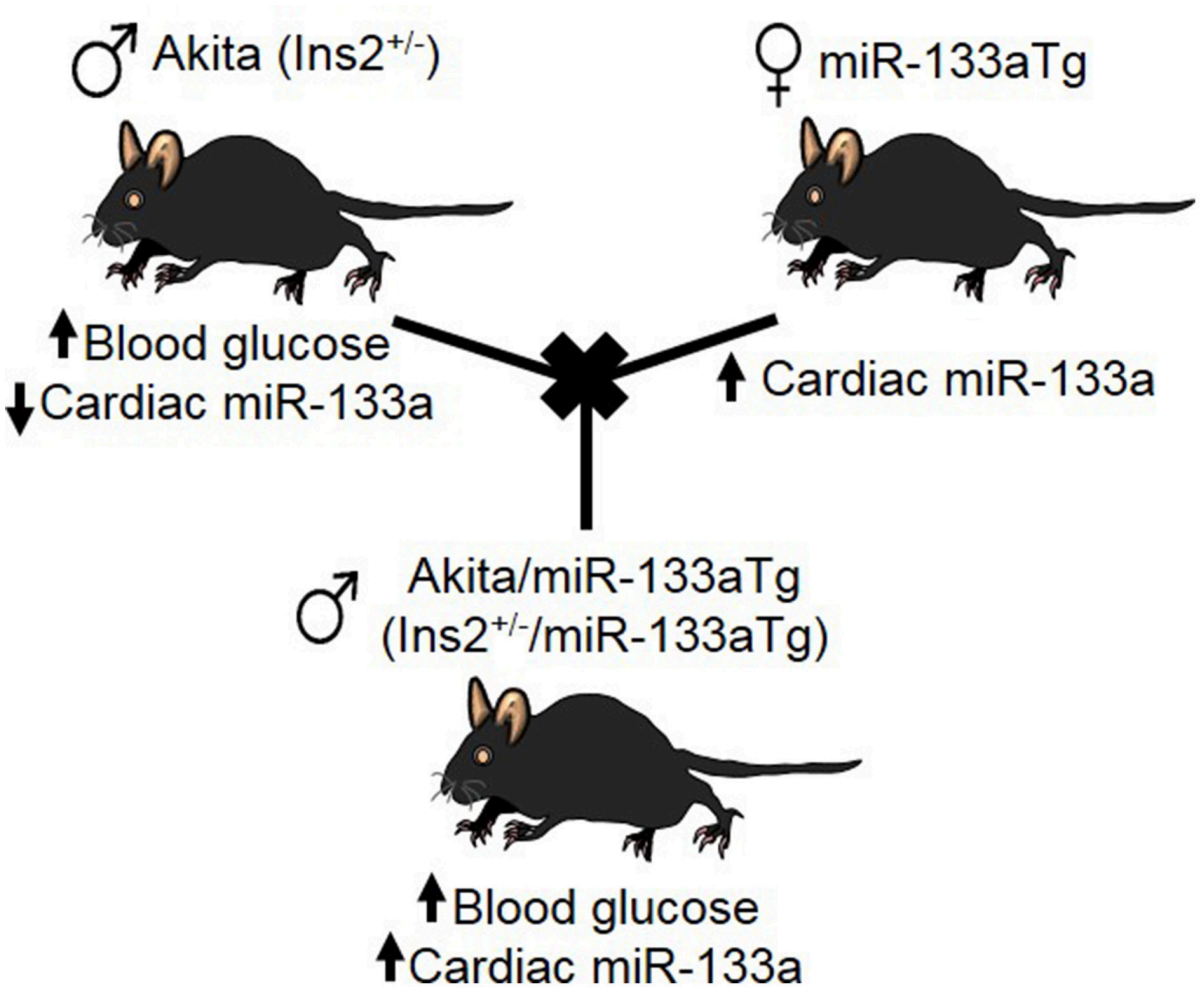
Creation of Akita/miR-133aTg mice. Schematic presentation of crossbreeding of Akita and miR-133aTg mice to create the novel strain of Akita/miR-133aTg mice.

Others and we have demonstrated that miR-133a is reduced in the DM heart (18, 19, 29, 30). Because miR-133a inhibits cardiac fibrosis (17) and hypertrophy (16), the key indicators of adverse cardiac remodeling, we sought to determine whether miR-133a over expression in the DM heart could prevent DM-induced cardiac remodeling. For this, we created a new strain of DM mice where miR-133a is upregulated in the heart, by crossbreeding Akita mice with cardiac-specific miR-133a transgenic (Tg) mice (Figure 1).

To rule out the effect of genetic background, we used Akita (spontaneous, genetic model of T1DM mice) and miR-133aTg mice with a C57BL/6J background. The Insulin 2 gene is present on chromosome 7, whereas the transgenic miR-133a gene is present on chromosome 18. The crossbreeding of Akita and miR-133aTg produces four F1 genotypes: WT (Ins2^+/+^), Akita (Ins2^+/−^), Akita/miR-133aTg (Ins2^+/−^/miR-133aTg), and miR-133aTg. We selected Akita/miR-133aTg mice for our studies (Figure 1).

### Validation of Akita/miR-133aTg Mice

**Figure 2:**
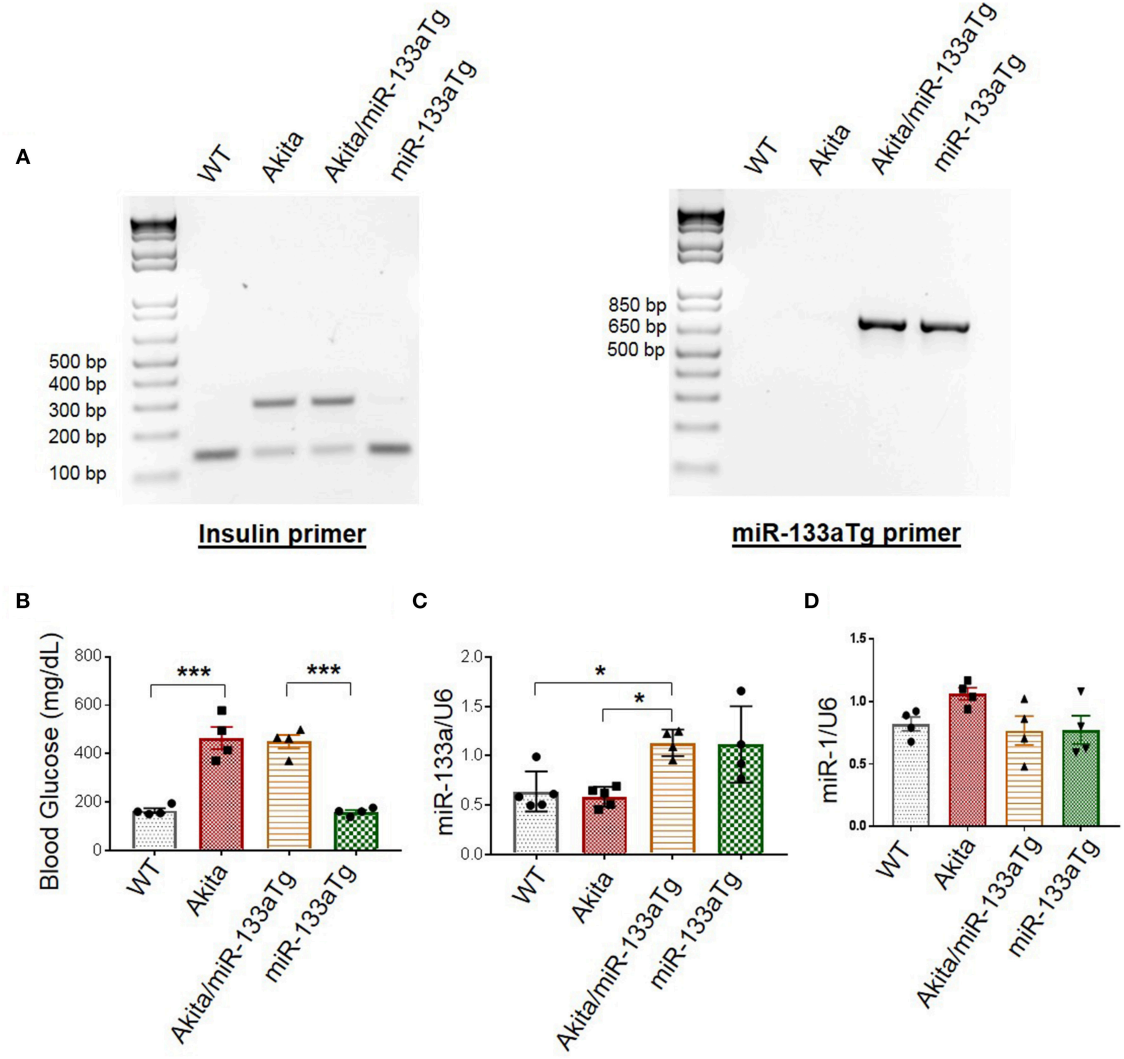
Validation of Akita/miR-133aTg mice. **(A)** Genotyping for WT, Akita, Akita/miR-133aTg, and miR-133aTg mice with Insulin 2 and miR-133aTg primers. There were two bands in Akita (Insulin 2 heterozygous) at 140 and 280 bp. There was one band for the miR-133aTg at 670 bp. **(B)** Blood glucose levels for WT, Akita, Akita/miR-133aTg, and miR-133aTg mice. There was no significant change in the blood glucose levels of Akita and Akita/miR-133aTg. **(C)** Gene expression levels of miR-133a isolated from the left ventricle of WT, Akita, Akita/miR-133aTg, and miR-133aTg hearts. **(D)** miR-1 quantification by individual miR-1 assay. Values are presented as mean ± SEM. One-way analysis of variance (ANOVA) followed by Tukey's test was used for statistical significance. *N* = 4–5 per group. *P < 0.05; ***P < 0.001.

To validate Akita/miR-133aTg mice, we first genotyped these mice. We used primers of Akita (Insulin primer) and miR-133aTg gene (miR-133a Tg primer) for polymerase chain reaction (PCR). Because Akita is heterozygous for insulin 2 gene, we found two bands of insulin at 140 bp (WT allele) and 280 bp (mutant allele). In WT mice, the mutant allele is absent and we found only one band representing insulin at 140 bp (Figure 2A). In miR-133aTg mice, we found only one band of miR-133aTg at 670 bp, which is absent in the WT mice (Figure 2A). In Akita/miR-133aTg mice, we found the genotypes of both Akita and miR-133aTg with the primers for insulin and miR-133aTg (Figure 2A).

Since Akita have DM, their blood glucose levels are high as compared to the WT (Figure 2B). We measured blood glucose levels in the four groups of mice: WT, Akita, Akita/miR-133aTg, and miR-133aTg. We found that blood glucose levels of Akita was comparable to Akita/miR-133aTg (Figure 2B). This suggests that miR-133a is not involved in reducing the levels of blood glucose in Akita mice.

We validated whether miR-133a is upregulated in the Akita/miR-133aTg heart by measuring miR-133a levels in the heart of the four groups of mice: WT, Akita, Akita/miR-133aTg, and miR-133aTg. We found ~2-fold increase in the levels of miR-133a in the Akita/miR-133aTg as compared to the Akita heart (Figure 2C). miR-133a is transcribed with miR-1 as a bicistronic transcript (31). We measured the levels of miR-1 in Akita/miR-133aTg heart and found no change in miR-1 levels (Figure 2D), suggesting that miR-1 level is not altered by miR-133a transgenic expression in the heart. These findings suggest that Akita/miR-133aTg mice have increased miR-133a in heart and it does not change cardiac miR-1 or blood glucose levels. Thus, our genotype and phenotype studies validate Akita/miR-133aTg as a new mouse strain of Akita where miR-133a is overexpressed in the heart.

### miR-133a Overexpression Prevents Lipotoxicity in the Akita Heart

**Figure 3:**
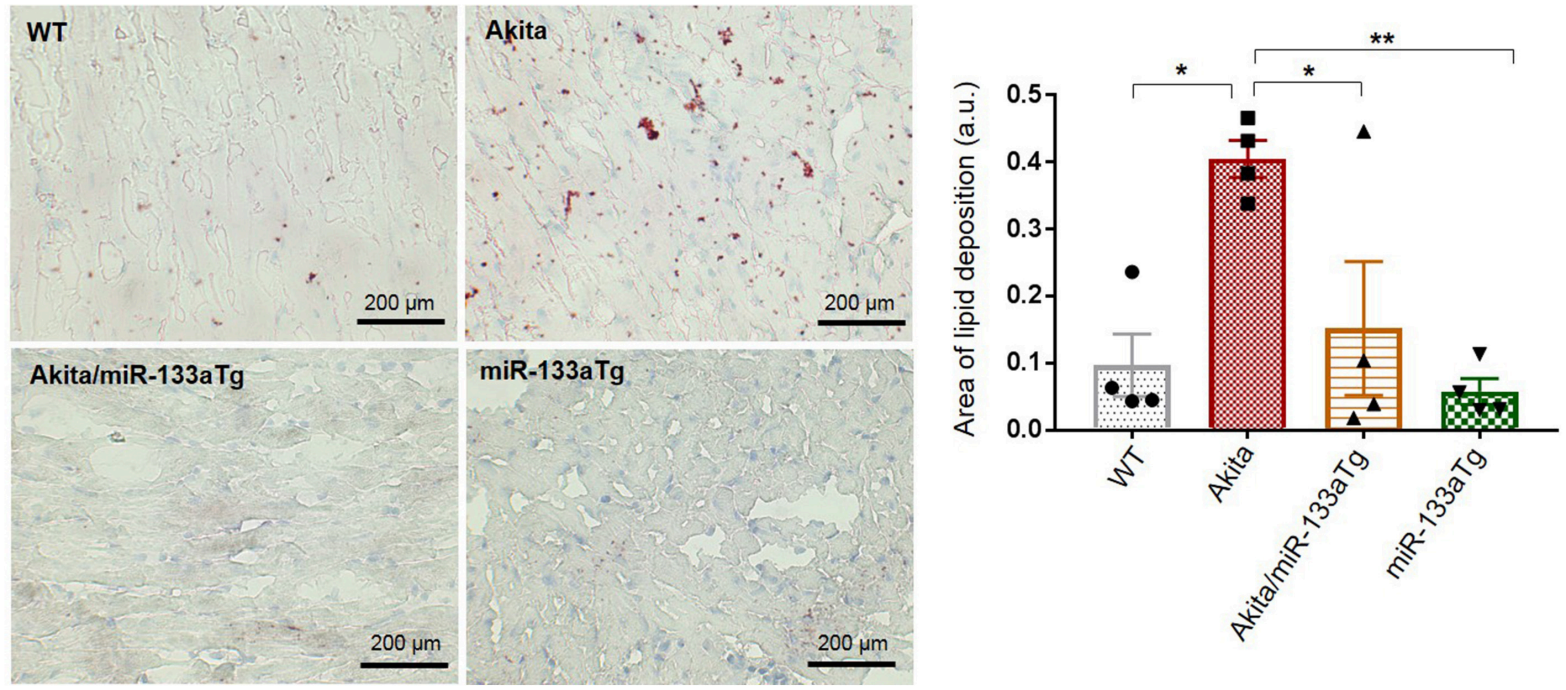
Cardiac-specific miR-133a prevents DM-induced lipid accumulation in the heart. Oil Red O staining of the heart cryosections. Representative left ventricle heart section of WT, Akita, Akita/miR-133aTg, and miR-133aTg mice (400× magnification). Quantification of red color in bar graph. Values are presented as mean ± SEM. One-way analysis of variance (ANOVA) followed by Tukey's test was used for statistical significance. Red color represents lipid deposition and light blue color represents nuclei. *N* = 4 per group. *P < 0.05; **P < 0.01.

Since overexpression of miR-133a in the Akita heart (Akita/miR-133aTg mice) did not have an impact on the elevated glucose levels, we sought to determine whether decreased miR-133a expression leads to metabolic remodeling. Previous reports have shown evidence of lipotoxicity in the Akita heart (32). To determine whether miR-133a prevents lipid deposits in the Akita heart, we stained the heart tissue sections of WT, Akita, Akita/miR-133aTg, and miR-133aTg with Oil Red O and quantified lipid deposits. We found increased lipid accumulation in the Akita heart; however, lipid accumulation was normal in the Akita/miR-133aTg heart (Figure 3). This result demonstrates that miR-133a prevents DM-induced lipotoxicity in Akita and could be involved in metabolic remodeling in the DM heart.

### Forced Expression of miR-133a in the Akita Heart Prevents Cardiac Remodeling by Blunting Cardiac Fibrosis and Hypertrophy

**Figure 4:**
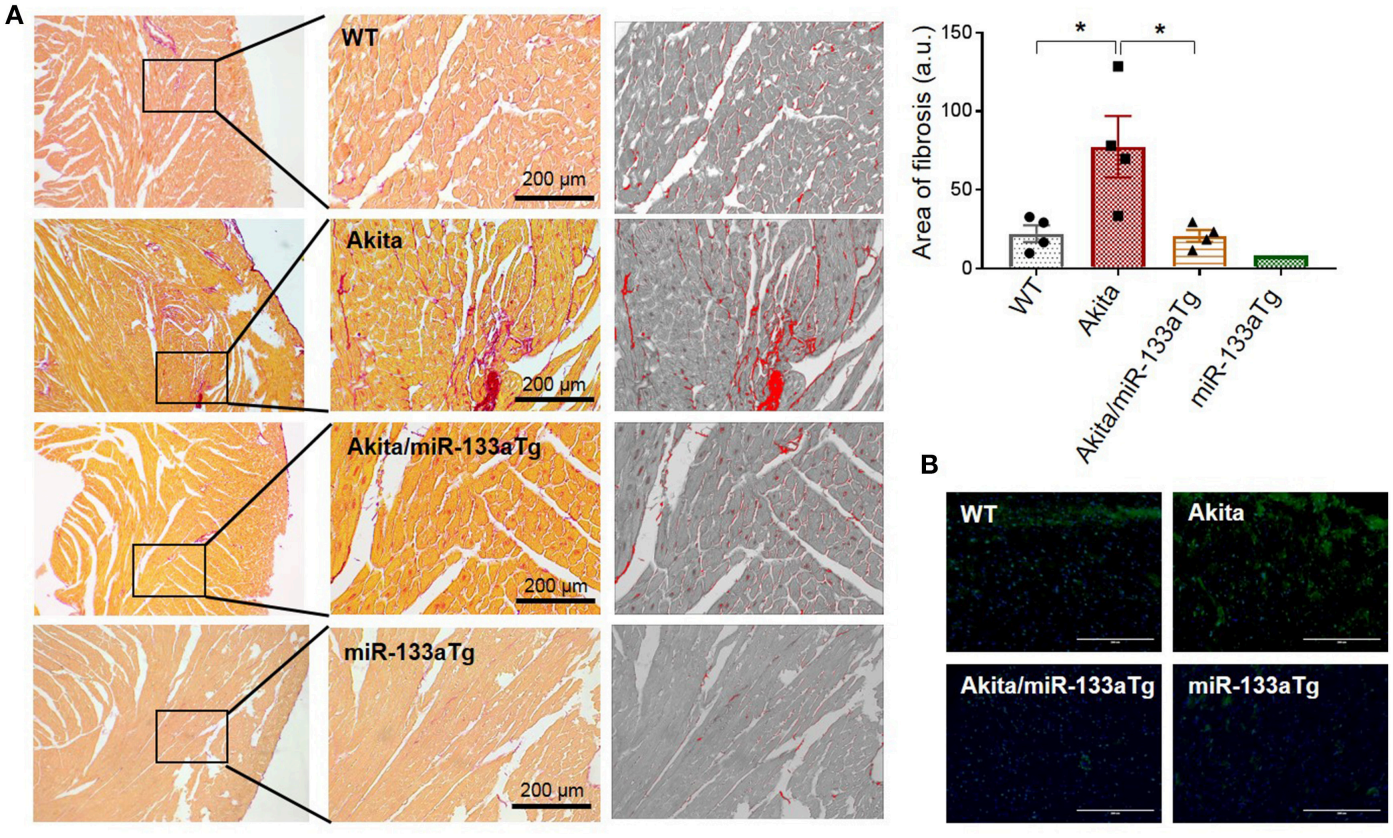
Cardiac-specific miR-133a prevents DM-induced cardiac fibrosis. **(A)** Picrosirius red staining of the heart from WT, Akita, Akita/miR-133aTg, and miR-133aTg mice. Values are presented as mean ± SEM. One-way analysis of variance (ANOVA) followed by Tukey's test was used for statistical significance. *N* = 4 per group. *P < 0.05. **(B)** *In situ* zymography for collagenase activity was performed on the heart sections of WT, Akita, Akita/miR-133aTg, and miR-133aTg mice. The green fluorescent protein represents cleaved collagen and blue color represents nuclei (DAPI staining). *N* = 1 per group.

Previous reports using miR-133aTg mice demonstrated that increased cardiac levels of miR-133a prevents pressure-overload-induced cardiac fibrosis (17). Transgenic miR-133a also prevents fibrosis (22) and remodeling (19) in the acute (streptozotocin-induced) T1DM heart. However, the effect of miR-133a overexpression in chronic DM and double DM was unclear. Here, we used Akita, which is a mouse model of chronic T1DM and double DM, to determine whether miR-133a overexpression in the Akita heart could prevent cardiac remodeling. In the heart, collagen I and collagen III are the major subtypes of collagen that contribute to cardiac fibrosis (33). We measured cardiac fibrosis by picrosirius red staining in the four groups of mice: WT, Akita, Akita/miR-133aTg, and miR-133aTg. Picrosirius red stains collagen content with red color, which can be quantified by measuring the red color's intensity (34). Our histological analysis of heart sections with picrosirius red staining showed increased collagen deposition in Akita and normalized collagen levels in Akita/miR-133aTg (Figure 4A). It suggests that increasing the levels of miR-133a could prevent DM-induced cardiac fibrosis in Akita. Cardiac fibrosis is associated with increased levels of matrix metalloproteinase-9 (MMP9), and inhibition of MMP9 mitigates cardiac fibrosis (35, 36). Thus, MMP9 is an important contributor of adverse cardiac remodeling (37, 38). We performed *in-situ* zymography to determine MMP9 activity in the histological sections of the heart. Our representative *in-situ* zymography showed increased MMP9 activity (increased intensity of green fluorescence) in Akita but less MMP9 activity in Akita/miR-133aTg (Figure 4B). These results demonstrate that miR-133a overexpression in the Akita heart prevents cardiac fibrosis.

**Figure 5:**
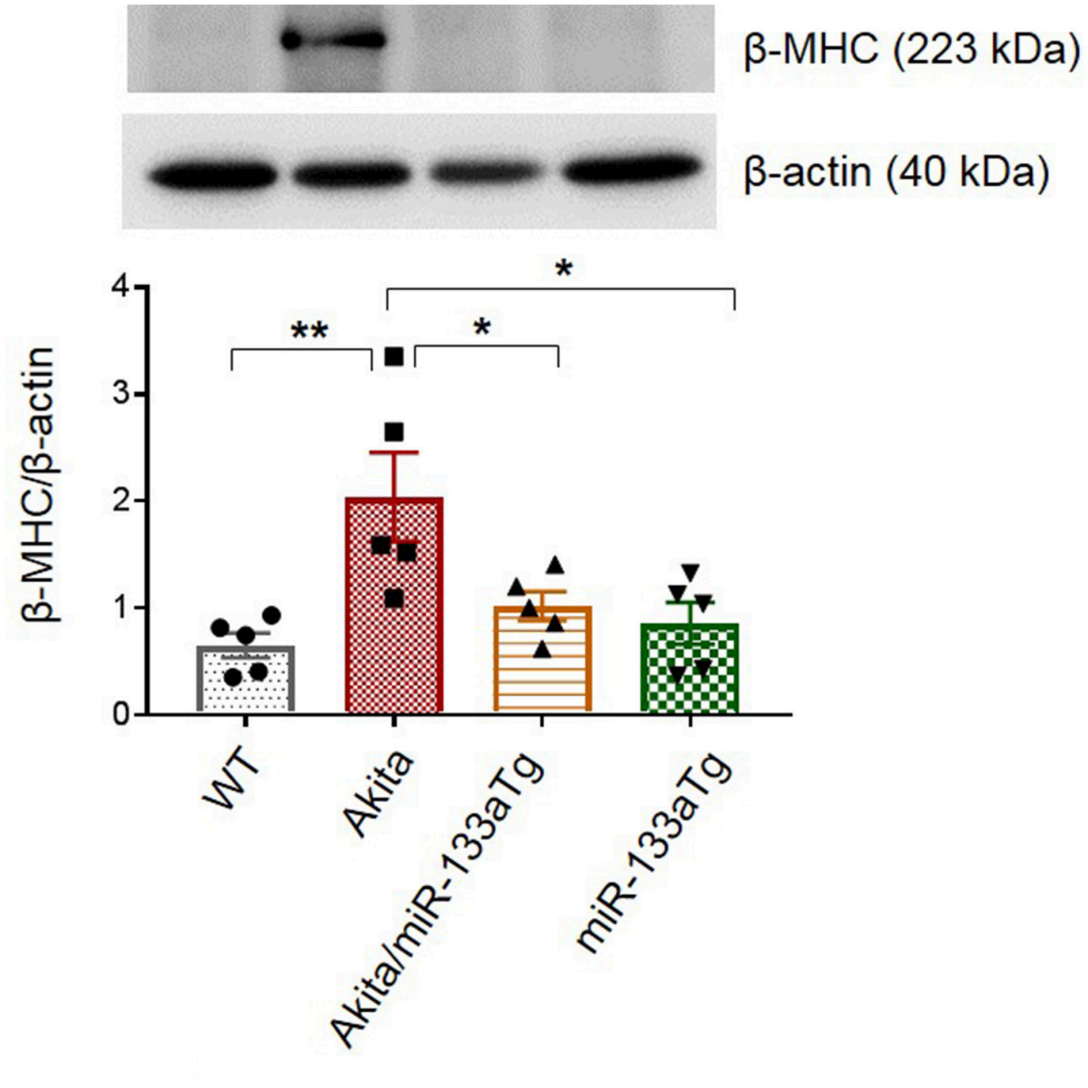
Overexpressed miR-133a blunts expression of β-MHC. Representative Western blot showing expression of beta-MHC and its densitometry analysis in the left ventricle of WT, Akita, Akita/miR-133aTg, and miR-133aTg mice. Values are presented as mean ± SEM. One-way analysis of variance (ANOVA) and Tukey's test were used. *N* = 5 per group. *P < 0.05; **P < 0.01.

Cardiac hypertrophy is an indicator of remodeling that is present in the Akita heart (29). To determine whether transgenic expression of miR-133a in the Akita heart could prevent cardiac hypertrophy, we measured the cardiac levels of beta-myosin heavy chain (β-MHC), a molecular marker of hypertrophy (39). Western blotting revealed that miR-133a overexpression blunts DM-induced cardiac hypertrophy in Akita (Figure 5). Altogether, these findings demonstrate that increased levels of miR-133a in the DM heart could prevent adverse cardiac remodeling.

### Increased miR-133a Levels in the Akita Heart Ameliorates Diabetic Cardiomyopathy

**Figure 6:**
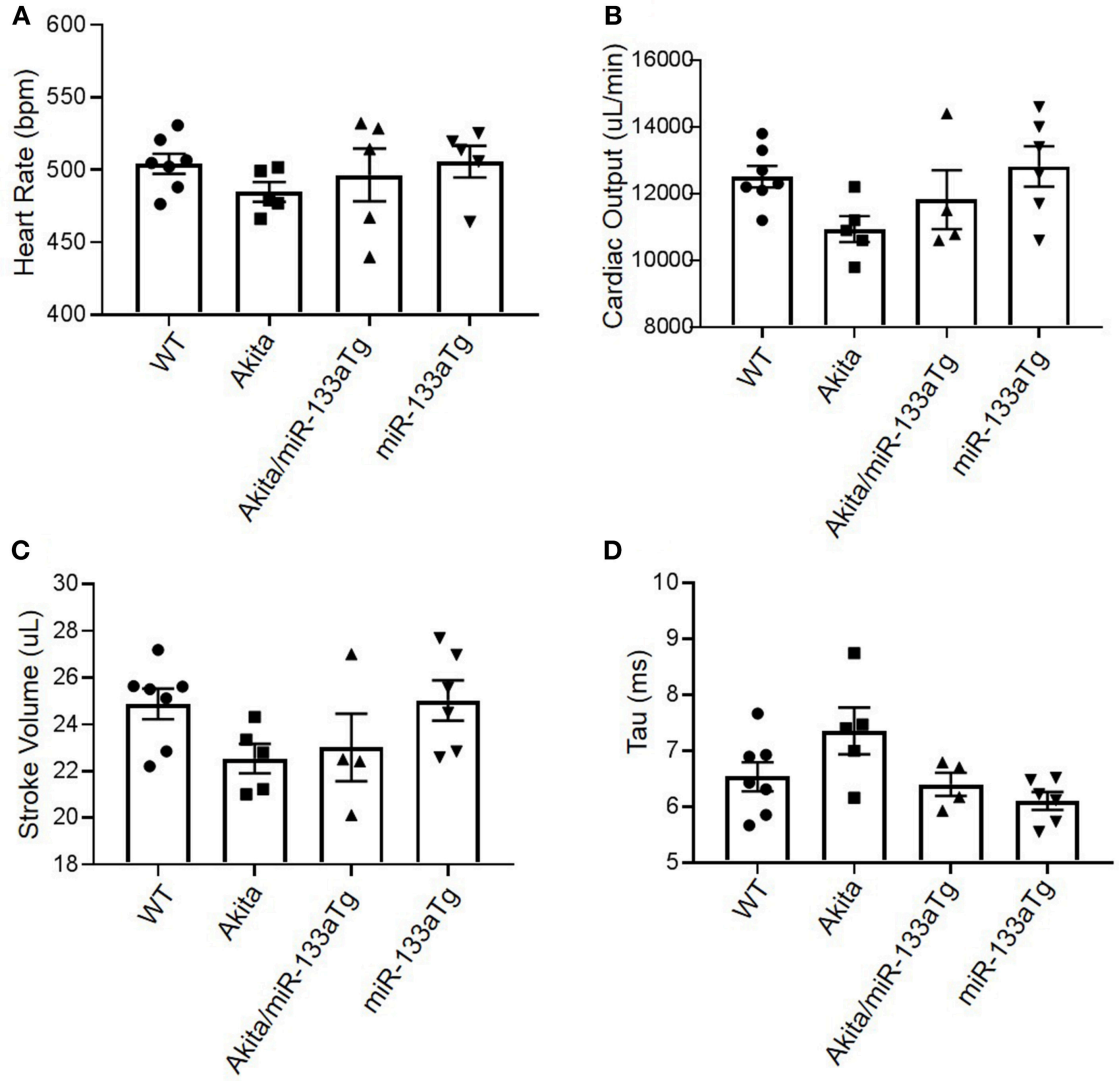
Pressure-volume (P-V) loop study for hemodynamic analysis. **(A)** The heart rate of mice from WT, Akita, Akita/miR-133aTg, and miR-133aTg group. **(B–D)** Cardiac output, stroke volume, and Tau in WT, Akita, Akita/miR-133aTg, and miR-133aTg mice. Values are presented as mean ± SEM. *N* = 4–7 per group. One-way analysis of variance (ANOVA) and Tukey's test were used.

To determine if transgenic expression of miR-133a in the Akita heart could prevent development of DMCM, we measured cardiac dysfunction by hemodynamic analysis using pressure-volume loop (20). We found relatively decreased cardiac output and stroke volume in Akita as compared to WT (Figures 6B,C), with comparable heart rates (Figure 6A). Interestingly, the trend of decreased cardiac output and stroke volume was blunted in Akita/miR-133aTg (Figures 6B,C). To determine impairment in diastolic relaxation, we measured Tau in these four groups. We found increased Tau in Akita indicating impaired active properties of diastolic relaxation. However, it was prevented in Akita/miR-133a (Figure 6D). These changes showed a trend and were not statistically significant suggesting that the 13–15 week Akita heart is in the progressing stage toward cardiac dysfunction.

**Figure 7:**
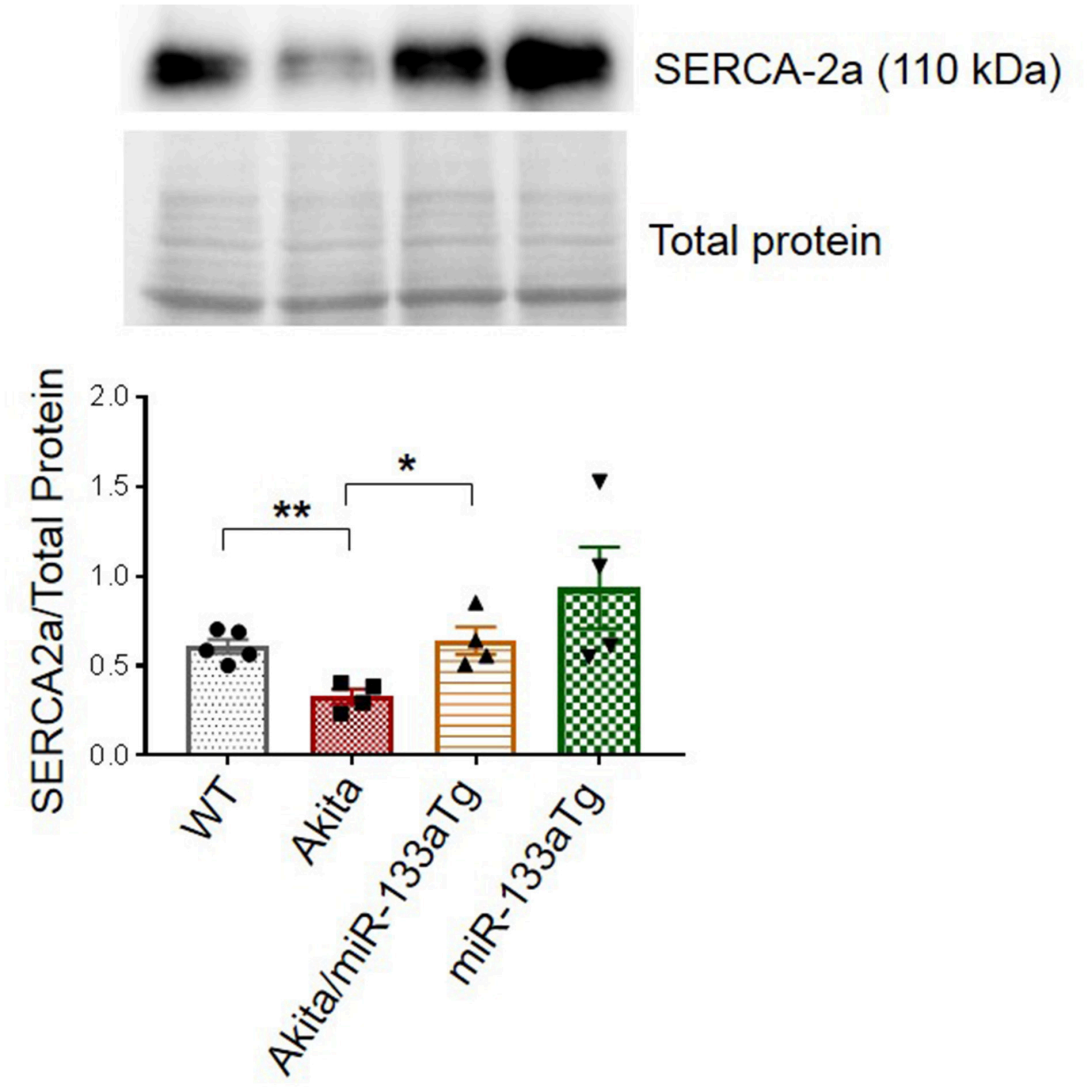
Overexpression of cardiac-specific miR-133a prevents loss of cardiac SERCA2a in Akita. Western blotting showing the representative SERCA2a protein bands and their densitometry analysis in the left ventricle of WT, Akita, Akita/miR-133aTg, and miR-133aTg mice. Values are presented as mean ± SEM. After one-way ANOVA, unpaired Student's *t*-test was used for statistical analysis. *N* = 4–5. ^*^P < 0.05; ^**^P < 0.001.

Since impaired contractility is a major contributor to DMCM and calcium handling protein sarcoendoplasmic reticulum calcium ATPase 2a (SERCA-2a) plays a key role in cardiac contractility, we measured the cardiac protein levels of SERCA-2a in these four groups of mice. We found decreased cardiac SERCA-2a in Akita as compared to WT. However, this decrease in SERCA-2a was prevented in Akita/miR-133aTg heart (Figure 7) suggesting that cardiac contractility is impaired at the molecular level in Akita and prevented by miR-133a transgenic expression in the heart. Altogether, these findings suggest that the forced expression of miR-133a in the Akita heart could prevent DMCM.

## Discussion

**Figure 8:**
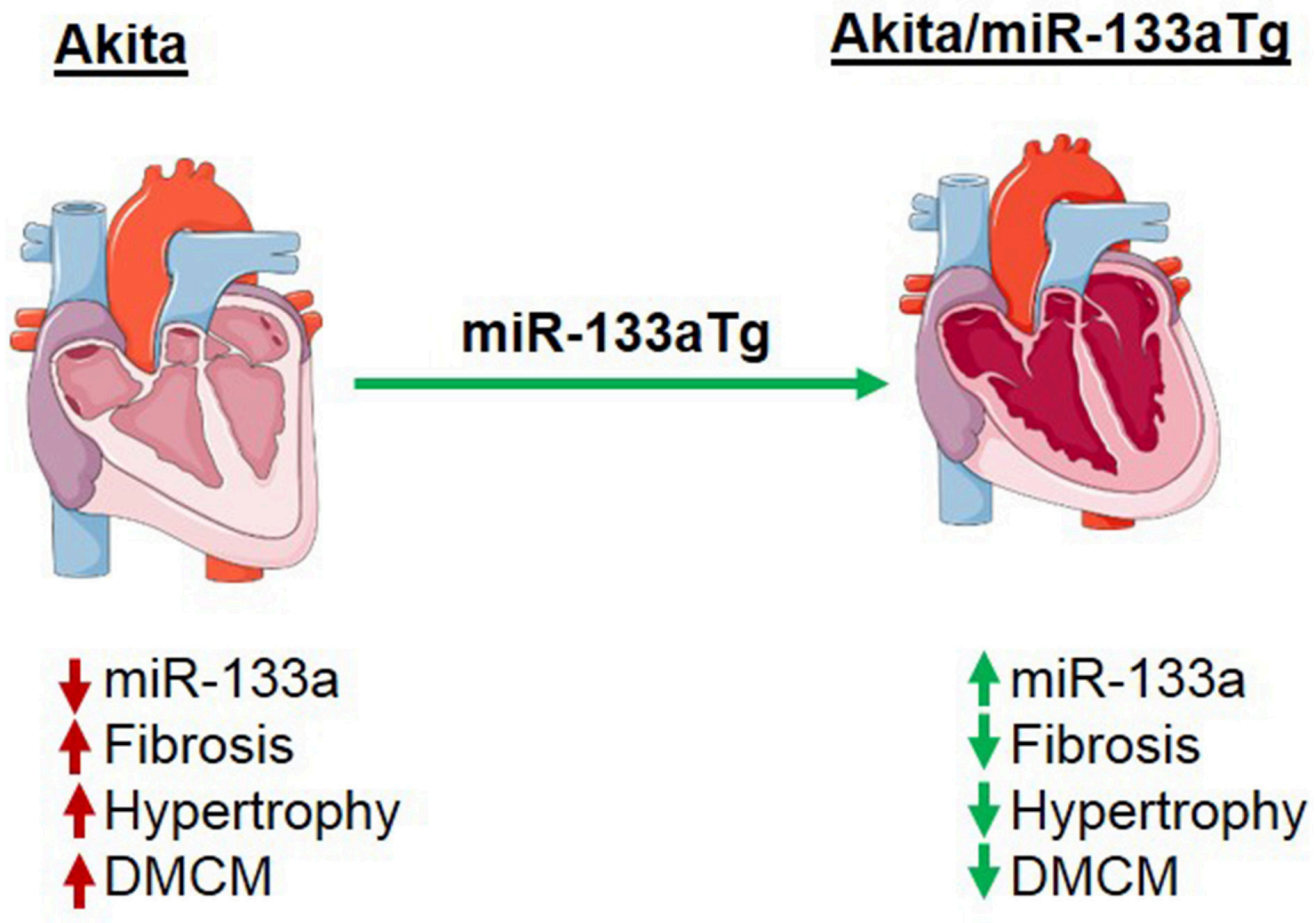
Schematic presentation showing the effect of miR-133a on prevention of cardiac fibrosis and cardiomyopathy in the diabetic heart. In the diabetic Akita heart, cardiac miR-133a is downregulated but cardiac fibrosis, hypertrophy, and diabetic cardiomyopathy (DMCM) were increased. However, transgenic expression of miR-133a in the diabetic Akita heart increases the levels of miR-133a and consequently decreases cardiac fibrosis, hypertrophy, and DMCM.

As DM increases in prevalence (40–42), the risk of DMCM and DM-induced heart failure poses a significant challenge. Currently used glycemic control strategy could deaccelerate the progression of DMCM (43) but it does not reduce the risk of heart failure in advanced stage of DM (6–8). Thus, investigating novel therapeutic strategies are warranted to prevent or cure DMCM. Our previous study revealed that miR-133a mimic treatment could mitigate cardiac dysfunction in the Akita heart (21). In the adult C57BL/6J mice, inhibition of only miR-133a by antigomiR causes cardiac hypertrophy (16). There are two forms of miR-133a: miR-133a-1 and miR-133a-2, with identical sequences located on chromosome 18 and chromosome 2, respectively (31). Deletion of any one form of miR-133a gene does not show any phenotype; however, deletion of both forms of miR-133a gene impairs cardiac development leading to embryonic lethality (31). Thus, miR-133a is required for cardiac development and for maintaining cardiac function in the adult heart (16, 31). The transgenic overexpression of miR-133a in the mouse heart does not show any phenotype and interestingly protects the heart against pressure-overload induced cardiac fibrosis (17). Thus, reduced levels of miR-133a is present in the pathological heart (16, 18) and increased cardiac levels of miR-133a do not have any detrimental effect (17). Since miR-133a is downregulated in the Akita heart, we sought to determine whether overexpressed miR-133a in the Akita heart could prevent adverse cardiac remodeling and cardiomyopathy. Our results revealed that transgenic expression of miR-133a in the Akita heart could stop DM-induced cardiac fibrosis, hypertrophy and DMCM (Figure 8). Thus, miR-133a could be a promising therapeutic candidate to prevent DMCM.

DMCM is a complex disease with far reaching etiologies (44). Restoring the regulatory signaling network could be a better therapeutic option to amend cellular homeostasis than targeting a particular gene that is upregulated or downregulated in the DM heart. miRNAs are evolutionary conserved endogenous regulators of the gene network, which control a majority of the biological signaling in our body by micromanaging the gene expression (10, 45). The heart is a highly regulated, complex, and vulnerable organ where subtle change in the regulation could have catastrophic effects. Thus, it is crucial to maintain the sophisticated regulatory network in the heart. miRNAs, through their ability to fine-tune gene expression, have emerged as a therapeutic target for cardiovascular diseases (12). Others (22, 30) and we (19, 21) have reported that miR-133a overexpression mitigates DMCM. However, it was unclear whether miR-133a overexpression could prevent cardiac remodeling and DMCM, which is crucial for pre-DM and early-stage DM patients to halt DMCM development. We created a new strain of Akita mice with miR-133a overexpressed in the heart (Akita/miR-133aTg) and validated them by genotyping and phenotyping. No change in the levels of miR-1 in Akita/miR-133aTg or miR-133aTg hearts suggest that miR-133a overexpression does not alters miR-1 level even though they are transcribed together as primary miRNA from bicistronic transcript (31).

In the present study, we used only male mice because female Akita mice do not have elevated levels of blood glucose and do not show an apparent DM phenotype. We used 13–15 weeks age group because Akita mice show increased blood glucose levels (250–300 mg/dL) at the age of 4 weeks and become robust hyperglycemic (>500 mg/dL) at the age of 8–12 weeks (20, 46). After hyperglycemia, it may take some time to initiate cardiac remodeling. We were interested to assess whether miR-133a could prevent early stage of cardiac remodeling, therefore, we selected 13–15 week mice where the heart shows cardiac fibrosis and hypertrophy. After initiation of cardiac remodeling, the heart gradually progresses toward cardiac muscle dysfunction or cardiomyopathy. Our results show a trend of decreased cardiac function in Akita. There was no significant difference, which was expected at 13–15 weeks of Akita. In older Akita (>20 weeks), we could observe significant cardiac dysfunction (21). However, this trend was blunted in Akita/miR-133aTg, suggesting that miR-133a transgenic expression in the Akita heart could prevent adverse remodeling and inhibits progression toward cardiac dysfunction. Notably, we found that at molecular level changes in the calcium handling protein SERCA-2a was significantly decreased in Akita and blunted in the Akita/miR-133aTg heart. It demonstrates that the cardiac dysfunction phenotype would be apparent in these Akita mice plausibly in the older age.

We found that overexpression of miR-133a in the Akita heart do not change blood glucose levels, suggesting that cardiac miR-133a is not involved in the regulation of glucose uptake. The comparable levels of miR-133a in Akita/miR-133aTg and miR-133aTg mice suggest that elevated levels of blood glucose do not affect miR-133aTg gene transcription or expression in the heart. We found that miR-133a blunts DM-induced lipotoxicity (32) in the Akita heart, which suggest that miR-133a could be involved in metabolic remodeling in the Akita heart. However, further studies are required to understand the underlying regulating mechanisms by which miR-133a prevents lipotoxicity in the Akita heart.

Although the protective effect of miR-133aTg against cardiac fibrosis is reported previously in a pressure–overload model (17), we show here that increased cardiac levels of miR-133a prevents DM-induced cardiac fibrosis. The expression of collagen in the Akita/miR-133aTg heart is comparable to the WT heart demonstrating that miR-133a overexpression may inhibit the DM-induced initiation of cardiac fibrosis. These results are corroborated by the MMP9 activity by *in-situ* zymography. MMP9 is a collagenase that degrades extracellular matrix (ECM) that causes new ECM synthesis and collagen build up leading to fibrosis. Thus, increased MMP9 activity is an indicator of fibrosis. We also show that miR-133a prevents the elevated expression of the early cardiac hypertrophy marker β-MHC (39), suggesting that increased levels of miR-133a could prevent hypertrophy in the DM heart. Our hemodynamic study utilizing pressure-volume loops revealed cardiomyopathy in Akita. Although statistical significance was not achieved, plausibly due to the early stage of cardiomyopathy, there was a trend of decreased stroke volume and cardiac output in Akita, which was improved in Akita/miR-133aTg mice. These results suggest that increased levels of miR-133a could resist against DM-induced cardiomyopathy. Notably, tau was relatively high in Akita while there was no change in the levels of tau in Akita/miR-133aTg. In addition, SERCA-2a was decreased in the Akita heart but there was no change in its level in the Akita/miR-133aTg heart. These findings suggest that miR-133a overexpression protects against DM-induced cardiac contractile dysfunction. These results support the previous finding that miR-133a mimic treatment improves contractility in streptozotocin-induced DM rats (19).

In summary, we have demonstrated for the first time that the forced expression of miR-133a in the DM heart could prevent cardiac remodeling and cardiomyopathy, plausibly by preventing metabolic and structural remodeling. These findings will set a platform to further test the effects of miR-133a overexpression in DMCM of other models of DM, as well as pre-DM and advanced-DM.

## Research Design and Methods

### Ethics Statement

All animal studies were performed following the guidelines of the National Institutes of Health and protocol approved by the *Institutional Animal Care and Use Committee* (*IACUC*) of the University of Nebraska Medical Center.

### Animal Model

We procured WT (C57BL/6J, stock # 000664) and Ins2^+/−^Akita (stock # 003548) mice from The Jackson Laboratory. The cardiac-specific miR-133aTg mouse strain was a gift from Dr. Scot Matkovich from the Washington University, St. Louis, USA. These mice were maintained in the animal care facility of the University of Nebraska Medical Center. They were provided with standard rodent chow and water *ad libitum*. We created Akita/miR-133aTg by crossbreeding Akita with miR-133aTg mice in the same animal care facility. We used 13–15 week old male mice for these studies.

### Glucose Measurement

Mice at early adulthood (8 weeks) were fasted for 7 h prior to glucose measurement. A small drop of blood was obtained from the tail and glucose was measured using an Accu-Chek Aviva glucometer (Roche Diagnostics, USA, catalog # 05963451001).

### Genotyping

A small piece of skin, obtained from ear punching, was used for DNA isolation. We isolated DNA using DNA isolation kit (Qiagen, Netherlands, catalog # 69506) and used 20 ng of DNA, Maxima Hot Start Green PCR Master Mix (Thermo Fisher Scientific, USA, catalog # K1061), and appropriate primers for polymerase chain reaction (PCR). For Insulin2 gene, the forward primer was 5′-TGCTGATGCCCTGGCCTGCT-3′, and the reverse primer was 5′-TGGTCCCACATATGCACATG-3′. The PCR reaction steps for Insulin 2 gene amplification were step 1, 94°C for 3 min, step 2, 94°C for 20 s, step 3, 64°C for 30 s, −0.5 C per cycle, step 4, 72°C for 35 s, step 5,repeat steps 2–4 for 12 cycles, step 6, 94°C for 20 s, step 7, 58°C for 30 s, step 8, 72°C for 35 s, step 9,repeat steps 6–8 for 25 cycles, step 10, 72°C for 2 min, and step 11, 4°C hold. The PCR product was incubated with Fnu4HI (Thermo Fisher Scientific, USA, catalog # ER1642) enzyme at 37°C for 3 h for restriction digestion before gel electrophoresis.

For miR-133aTg PCR, the forward primer was 5′-GAAGC CTAGCCCACACCAGAAATG-3′ and the reverse primer was 5′-TGAGTAAGTTGAGATGTAATTCATGCTCA-3′. The PCR steps for miR-133aTg amplification were step 1, 94°C for 5 min, step 2, 94°C for 1 min, step 3, 53°C for 1 min, step 4, 72°C for 1 min, Step 5, repeat steps 2–4 for 30 cycles, Step 6, 72°C for 5 min, and step 7, 4°C hold.

We performed PCR reaction on C1000 Touch Thermal Cycler (Bio-Rad Laboratories, USA, catalog #1851148). The PCR product was used for gel electrophoresis with a 1.5% agarose gel. We imaged the gel bands using ChemiDoc XRS system (Bio-Rad Laboratories, USA, catalog # 1708265).

### RNA Extraction and miRNA Assays

Total RNA was extracted from the left ventricle (LV) of the heart using mirVana miRNA isolation kit (Thermo Fisher Scientific, USA, catalog # AM1560). Measured by a NanoDrop One (Thermo Scientific Inc., USA, catalog # ND-ONE-W), high quality RNA (260/280 > 1.8) was used for miR-133a assay. We converted RNA into cDNA by using TaqMan® microRNA reverse transcription kit (Life Technologies, USA, catalog # 4366597). We amplified miR-133a by using TaqMan primers for miR-133a (assay ID: 002246), miR-1 (assay ID: 002222), and the endogenous control U6 snRNA (assay ID: 001973) from Thermo Fisher Scientific, USA, and using CFX Connect Real-Time PCR Detection System (Bio-Rad Laboratories, USA, catalog # 1855201). We analyzed the qPCR results by CFX Manager3.0 software (Bio-Rad Laboratories, USA).

### Picrosirius Red Staining

We used the UNMC Tissue Sciences Facility to perform histological staining. We used 5 μM transverse paraffin sections of the heart, fixed them for Picrosirius red staining. The red color represents collagen accumulation in the heart. We acquired images using a VWR bright field microscope (VWR, USA, catalog # 89404-890), selected the same areas of tissue in the left ventricle and compared them in all groups. We used NIH Image J software for quantification of the fibrotic area in different groups.

### Oil Red O Staining

Transverse heart cryosections were air-dried before fixing with 10% formalin for 5 min. They were then air-dried again for 60 min and washed in 60% isopropyl alcohol for 5 min. We prepared Oil Red O working solution and filtered it to remove precipitates [0.5% Oil Red O (Sigma, USA, catalog # O0625-25G) dissolved in isopropyl alcohol (W/V)]. These solutions were applied on the heart sections for 15 min. The sections were then washed with 60% isopropyl alcohol for 5 min, dipping the slides in and out 3x. We counter stained them with hematoxylin for 1 min, washed 3x in water, and then mounted with a cover glass and fluoromount aqueous mounting solution for imaging under a bright field microscope. The intensity of color (red) was quantified by Image J software.

### *In-situ* Zymography

We obtained the heart cryosections by using the CryoStar NX50 (Thermofisher scientific, USA). Collagenase activity was measured using the Enzcheck Gelatinase/Collagenase Assay kit (Molecular Probes, USA catalog # E-12055) following the kit protocol. For staining the nucleus, we first permeabilized the sections with 0.1% Triton X and after washing, stained with 1 ug/ml DAPI (AppliChem, USA catalog # A1001) dissolved in PBS for 20 min. We observed green fluorescence for MMP9 activity and blue fluorescence for nuclei (DAPI) by using the EVOS FL Imaging System (Life Technology, USA, catalog # AMF 4300).

### Pressure-Volume Loop Study

We measured hemodynamic changes in the heart by pressure-volume (P-V) loop study using ADV500 system, Transonic Scisense, USA (20). We followed the best practice guidelines for invasive hemodynamic measurement in mice described by Lindsey et al. (47). We anesthetized mice with 1–2% isoflurane, intubated it and connected it to a ventilator (MidiVent, Harvard Apparatus, USA). Then, we opened the chest and created a fine hole in the apex area of left ventricle (LV) using a 27-gaug needle and immediately inserted a 1.2F 4.0 mm catheter probe (Transonic Scisence, USA). The probe was advanced slowly into the LV in order to avoid contact with the LV and septal walls, which was ensured by observing the P-V loop formation. Since body temperature may contribute to the hemodynamic measurements, we maintained the body temperature at 37°C by a temperature controller (ATC2000, World Precision Instruments, USA). Admittance technique and Wei's equation were used to calculate volume (48). Because heart rate could affect hemodynamic changes, we kept the heart rate between 450 and 500 beats per minute by adjusting anesthesia. Hemodynamic parameters of diastolic function were determined by averaging at least 20 cardiac cycles following a baseline scan, which was performed after proper probe placement was confirmed using the LabChart Pro PV Loop Analysis Add-On.

### Protein Extraction and Western Blotting

Protein was extracted from the LV tissue of the heart using Radio-Immunoprecipitation Assay Buffer or RIPA (Boston BioProducts, USA, catalog # BP-115) and protease and phosphatase inhibitor (Sigma-Aldrich, USA, catalog # MS-SAFE). The protein lysate was centrifuged at 10,000 g at 4°C for 12 min. The supernatant was collected and pellet was discarded. Protein concentration was estimated by using Pierce BCA Protein Assay Kit (Thermo Scientific Inc., USA, catalog # 23225). For Western blotting, 25 μg protein was loaded in the wells of a 12% polyacrylamide gel for electrophoresis. The gel was then transferred onto a nitrocellulose membrane (Bio-rad Laboratories, UA, catalog # 1620115). We performed Ponceau staining (Thermo Scientific, USA, catalog # BP103-10) of the transferred membrane, imaged it in ChemiDoc XRS system (Bio-Rad Laboratories, USA) and quantified it for total protein estimation. We blocked the membrane using non-fat dry milk (Bio-rad Laboratories, catalog #170-6404) in 5% milk in Tris-buffered saline (TBS) at room temperature for 1 h. We incubated the membrane with SERCA2a (Abcam, USA, catalog # ab2861) primary antibody (1:1,000 dilution in TBS) and β-MHC primary antibody (Abcam, USA, catalog #ab50967) overnight at 4°C. After washing the membrane (3 min for three times), we incubated the membrane with anti-mouse IgG-HRP (Cell Signaling Technology, USA, catalog #7076) at a dilution of 1:2,000 in TBS for 2 h at room temperature. The membrane was developed in a chemiluminescence substrate (Bio-Rad Laboratories, USA, catalog #170-5061) using a ChemiDoc XRS system (Bio-Rad Laboratories, USA) and the densitometry analysis of the bands was done by the Image Lab 4.1 software (Bio-Rad Laboratories, USA).

### Statistical Analysis

We presented values as the mean with standard error of the mean (mean ± SEM). To compare the means from all four groups, we used one-way analysis of variance (ANOVA) followed by Tukey's *post-hoc* test unless stated otherwise. To compare means of two groups, we used Student's *t*-test. For statistical analysis, we used GraphPad Prism Version 7.02 software from GraphPad Software Inc., San Diego, USA.

## Author Contributions

TK and HS coordinated and performed majority of experiments, analyzed data, and wrote manuscript. SK and SY contributed to experiments and analysis of experimental results, contributed in writing, and editing the manuscript. PM conceived and supervised the study, corrected the manuscript, and approve the final version of the manuscript.

### Conflict of Interest Statement

The authors declare that the research was conducted in the absence of any commercial or financial relationships that could be construed as a potential conflict of interest.
